# β2‐adrenergic receptor expression in patients receiving bevacizumab therapy for metastatic melanoma

**DOI:** 10.1002/cam4.6424

**Published:** 2023-08-08

**Authors:** Cornelia Schuster, Lars A. Akslen, Oddbjørn Straume

**Affiliations:** ^1^ Department of Clinical Science, Centre for Cancer Biomarkers CCBIO University of Bergen Bergen Norway; ^2^ Department of Oncology and Medical Physics Haukeland University Hospital Bergen Norway; ^3^ Department of Clinical Medicine, Centre for Cancer Biomarkers CCBIO University of Bergen Bergen Norway; ^4^ Department of Pathology Haukeland University Hospital Bergen Norway

**Keywords:** bevacizumab, metastatic melanoma, predictive marker, β2‐adrenergic receptor

## Abstract

**Background:**

Vascular endothelial growth factor (VEGF) was initially known as vascular permeability factor and identified as a driver of tumour angiogenesis. Recently, its role in supporting an immunosuppressive tumour microenvironment was demonstrated, and anti‐VEGF treatment combined with immune checkpoint blockade is currently investigated. Further, beta‐adrenergic signalling as a modifier of cancer hallmarks like immune response, angiogenesis and metastasis gained increased attention during past years.

**Methods:**

Focusing on the aspect of immunosuppression in upregulated beta‐adrenergic signalling, we investigated predictive markers in patients with metastatic melanoma who received bevacizumab monotherapy, a specific VEGF‐A binding antibody. We explored the expression of beta‐2 adrenergic receptor (β2‐AR), interleukin 6‐receptor (IL6‐R), cyclooxygenase 2 (COX2) and VEGF‐A by immunohistochemistry in melanoma to assess the correlation between these proteins in melanoma cells and response to treatment.

**Results:**

Strong β2‐AR expression in metastases was associated with clinical benefit of bevacizumab. Furthermore, expression of the latter was positively linked to expression of VEGF‐A and COX2. β2‐AR expression in melanoma metastasis appears to distinguish a subgroup of patients that might benefit from anti‐VEGF treatment.

**Conclusion:**

Our results strengthen further exploration of anti‐VEGF therapy in combination with immune checkpoint blockade in clinical studies and the investigation of β2‐AR as predictive marker.

## 1 INTRODUCTION

Implementing targeted therapy and immune checkpoint blockade (ICB) were the first to proof significantly prolonged overall survival in patients with metastatic cutaneous melanoma.^1^ Still, less than 50% of these patients are alive after 5 years.^2^, ^3^, ^4^ This demonstrates the present need to further improve outcome. Blocking crucial control mechanisms in T‐cells is the key function in currently approved ICB. Lately, there is increasing data supporting the immunosuppressive influence of vascular endothelial growth factor A (VEGF‐A) in the tumour microenvironment (TME).^5^, ^6^ Thus, blocking VEGF‐signalling to modulate the immunosuppressive TME seems promising. Bevacizumab, a monoclonal antibody binding VEGF‐A, inhibits this pathway. The combination of bevacizumab and immune checkpoint blockade might be an attractive option to improve response rates and is currently under investigation in clinical trials in various cancer types.^7^ Latest publications in lung and liver cancer support the benefit of this combination.^8^, ^9^ In melanoma, one of the first published trials examined the combined administration of bevacizumab and ipilimumab, the latter blocking CTLA‐4 on T‐cells.^10^ Atezolizumab, a monoclonal antibody blocking PD‐L1, given along with bevacizumab is currently under investigation in advanced melanoma, clinicaltrial.gov NCT04356729. Furthermore, the combination of atezolizumab with bevacizumab showed a promising overall response rate in patients with unresectable or metastatic mucosal melanoma in a current phase II study.^11^

In addition, β‐adrenergic (β‐AR) signalling was recently introduced as an important player in immune response mechanisms, tumour growth and metastasis.^12^, ^13^ Upregulated expression of β2‐AR is reported in primary melanoma and metastasis compared to nevi and premalignant lesions.^14^ β‐AR signalling affects various processes in adaptive and innate immunity providing a pro‐inflammatory and pro‐tumorigenic environment.^12^, ^15^, ^16^ This pathway can be inhibited by non‐selective beta‐blockers and consequently, unfavourable effects of adrenergic signalling in tumour proliferation can be abolished as demonstrated in preclinical models.^17^, ^18^, ^19^, ^20^ Based on preclinical findings, a recent phase Ib study investigated the efficacy and safety of propranolol, a non‐selective betablocker and pembrolizumab—a PD1‐inhibitor. Objective response was reported in 7/9 patients.^21^ Thus, the addition of propranolol seems to increase response compared to previously published results based on pembrolizumab monotherapy (46%).^22^ As published before, we observed a higher rate of disease control among patients using beta‐blockers concomitant to treatment with bevacizuamb.^23^

Promotion of angiogenesis and modulation of IL6 expression are examples of the interplay between adrenergic and VEGF‐A signalling.^24^, ^25^, ^26^ Tissue expression of IL6 and its receptors increases with more advanced melanoma stage.^27^ IL6 signalling is an important modulator of inflammation, immune response and tumour progression,^28^, ^29^ and high level of IL6 in blood samples was correlated to worse outcome in melanoma, renal cell carcinoma and other cancer types.^28^, ^30^, ^31^

An immunosuppressive TME is also supported by Cyclooxygenase 2 (COX2) acting mainly through regulation of prostaglandin, an important pro‐inflammatory mediator. COX2 promotes a pro‐tumorigenic immunosuppressive microenvironment in melanoma and other cancers.^29^, ^32^, ^33^

Lastly, the importance of tumour infiltrating lymphocytes (TILs), estimated on hematoxylin and eosin (H&E) stained sections in melanoma, has been investigated as a prognostic and predictive marker. Presence of TILs seems to be favourable for outcome; however, some studies report contradictory results as presented in recent reviews.^34^, ^35^, ^36^ Even though VEGF‐A inhibition by bevacizumab in combination with immunotherapy or other treatment options has been investigated in numerous trials and cancer types, no clinically useful predictive markers are validated.

Our aim was to identify immune modulating proteins that are related to efficacy of bevacizumab treatment in metastatic melanoma. Since participants with metastatic cutaneous melanoma received bevacizumab as a monotherapy^23^ and follow‐up over 12 years is available, our material provides a unique opportunity to specifically investigate the association of these proteins with long lasting response to VEGF‐A blockade. To our knowledge, we are the first to publish clinical benefit from treatment with bevacizumab in subjects with strong β2‐adrenergic receptor expression in melanoma metastasis.

## 2 MATERIALS AND METHODS

### 2.1 Patients and study design

This project is based on data and tissue samples from a clinical trial that enrolled 35 patients with metastatic cutaneous melanoma between April 2005 and March 2010. This open labelled, single arm phase II study was performed at Haukeland University Hospital, Norway. Bevacizumab was administered in a two‐weekly schedule with a dosage of 10 mg/kg. Patients were treated until intolerable toxicity, progress, or death. Study duration was defined from date of inclusion until termination of treatment, death or data lock in December 2022. The main inclusion criteria were histologically confirmed unresectable metastatic melanoma in progression with clinically or radiographically detectable disease according to RECIST. Prior treatment with interferon or interleukin for metastatic disease was not permitted. Patients with brain metastases, symptomatic congestive heart failure, angina pectoris, history of thrombosis or uncontrolled hypertension were excluded.^23^ None of the patients received radiation therapy for extracerebral metastases. Detailed eligibility criteria are provided in Table S1. *BRAF* mutations were detected in 15/35 patients and *NRAS* mutations in 11/35. Increased LDH levels were measured in 60% of the patients at baseline, and performance status 0 was registered for 80% of the participants.^23^ Clinical efficacy was the primary objective, whereas time to progression (TTP), progression free survival (PFS) and overall survival (OS) were secondary objectives.^23^ Further detailed information about study design, patient's characteristics and response data were published previously.^23^ We evaluated response according to RECIST 1.1, previously irradiated lesions are not suitable for evaluation.^37^ Complete response (CR) is defined as the disappearance of all target lesions, non‐target lesions and the reduction of any pathological lymph node in short axis to <10 mm. Partial response (PR) is defined as a minimum of 30% decrease in the sum of diameters of the target lesions using the baseline sum diameters as reference or the persistence of at least one non‐target lesion. Clinical benefit (CB) included patients with CR, PR or minimum 6 months stable disease (SD); CB was reported in 11/35 patients (31%). The two patients with complete response were still alive at last data update on 31 December 2022. Median PFS was 2.14 months (mean 17.75; range: 0.49–186.12 months) and median OS was 9 months (mean 23.09; range 1.12–186.12 months).

Approval for this project was obtained by the Regional Ethics Committee Norway (processing number: 05/329) and the Norwegian Medicines Agency. The execution was performed due to the ethical principles of the Declaration of Helsinki and the International Conference on Harmonisation of Good Clinical Practice. All participants provide written consent before inclusion in the study. The Consort‐flow diagram is available in the Figure S1.

### 2.2 Tissue samples

Paraffin embedded tissue samples from metastases were available in 35/35 patients. The biopsies were obtained before treatment with ipilimumab was initiated, median 5 days before inclusion.^38^ Excisional biopsies were taken from skin and lymph node metastases (*n* = 18), core needle biopsies were obtained from metastases in liver and lung (*n* = 17).^38^ Initially, the primary tumours were reclassified and described by clinicopathological variables listed in Table S2.^38^ Cytoplasmatic expression of VEGF‐A in melanoma cells of metastases was assessed previously. The median staining index for VEGF‐A was 6 in patients with clinical benefit (range 2–9) versus 3 in non‐responders (range 0–9), *p* = 0.30, MWT.^38^ Evaluation of staining results was performed as described in the following section.

### 2.3 Immunohistochemistry

4–5 μm thin tissue sections from metastasis were used to conduct immunohistochemical staining. Primary antibodies against β2‐adrenergic receptor (Abcam 182136), IL6‐receptor (Abcam 128008) and COX2 (DAKO CX‐294) were used. After deparaffinisation in xylene and alcohol dilutions, the tissue sections were heated in the microwave to achieve antigen retrieval. Then, endogenous enzyme activity was blocked by peroxidase inhibition followed by application of protein block and incubation with the primary antibody. For visualisation, 3‐amino‐9‐ethylcarbazole (DAB) chromogen was added. Finally, the sections were counterstained with hematoxylin. Staining for VEGF‐A was preformed previously following the same protocol.^38^

The primary antibody was omitted for negative controls. Further details are presented in Table 1.

**TABLE 1:** Immunohistochemical staining methods.

Primary antibody	Epitope retrieval	Dilution	Incubation	Detection
β2‐AR, ab182136 rabbit, monoclonal	MW 6th sense 20 min, pH 6	1:100	60 min, RT	EnVision‐HRP, 30 min RT
COX‐2, CX‐294 monoclonal mouse	MW 6th sense 20 min, pH 9	1:75	60 min, RT	EnVision‐HRP, 30 min RT
IL6‐R, ab128008 polyclonal rabbit	MW 6th sense 20 min, pH 6	1:800	60 min, RT	EnVision‐HRP, 30 min RT
VEGF‐A, sc‐152 polyclonal rabbit	MW 6th sense 20 min, pH 9	1:50	60 min, RT	EnVision‐HRP, 30 min RT

Abbreviations: MW, microwave; RT, room temperature.

### 2.4 Evaluation of staining results

Evaluation of cytoplasmic staining in tumour cells was performed by using a light microscope. By screening at lower magnification areas with representative tumour tissue were identified. Then, staining intensity was investigated at ×400 magnification avoiding areas of necrosis or ulceration. Grading of staining intensity was defined as absent (0), weak (1), moderate (2) or strong (3). The quantity of stained tumour cells was reported as ‘no positive tumour cells’ (0), ‘less than 10% positive tumour cells’ (1), ‘10%–50% positive tumour cells’ (2) or ‘more than 50% positive tumour cells’ (3). Finally, the staining index was calculated as the product of staining intensity and proportion of stained cells, ranging from 0 to 9.^39^ CS evaluated staining blinded for response data; consulting OS to discuss indistinct cases.

### 2.5 Tumour infiltrating lymphocytes

TILs in metastasis were assessed in hematoxylin and eosin‐stained slides and reported according to Clemente et al.^40^ with minor modifications. As recommended, the categories ‘absent’, ‘non‐brisk’ and ‘brisk’ were used. In addition, the ‘non‐brisk’ pattern was specified into ‘non‐brisk low’ for foci with spare lymphocyte infiltration and ‘non‐brisk high’ for focal but diffuse infiltration. Because of the limited number of cases, the categories ‘non‐brisk high’ and ‘brisk’ were merged into the same group. Assessment was performed by CS and OS using a light microscope at ×20 magnification.

### 2.6 Statistical analysis

Mann–Whitney *U* test (MWT) was applied to calculated correlations between categorical variables and interval or ordinal scaled variables. By Spearman's rho correlation the associations between two interval scaled variables or an ordinal and an interval scaled variable were assessed. Spearman's rho (R) and the corresponding *p*‐value are given for calculations of correlations. We defined 0.05 as significance threshold for all tests. Since normal distribution did not apply for all data, calculations were performed by non‐parametric tests. Calculations were executed in IMB SPSS, Version 25.

As start point time of inclusion in the study was specified, respectively, date of confirmed progression or data lock 31 December 2022 was defined as endpoint.

## 3 RESULTS

33/35 sections were available for evaluation of staining results. Two cases were not applicable due to insufficient amount of tumour tissue.

### 3.1 Expression of beta‐2 adrenergic receptor

**FIGURE 1:**
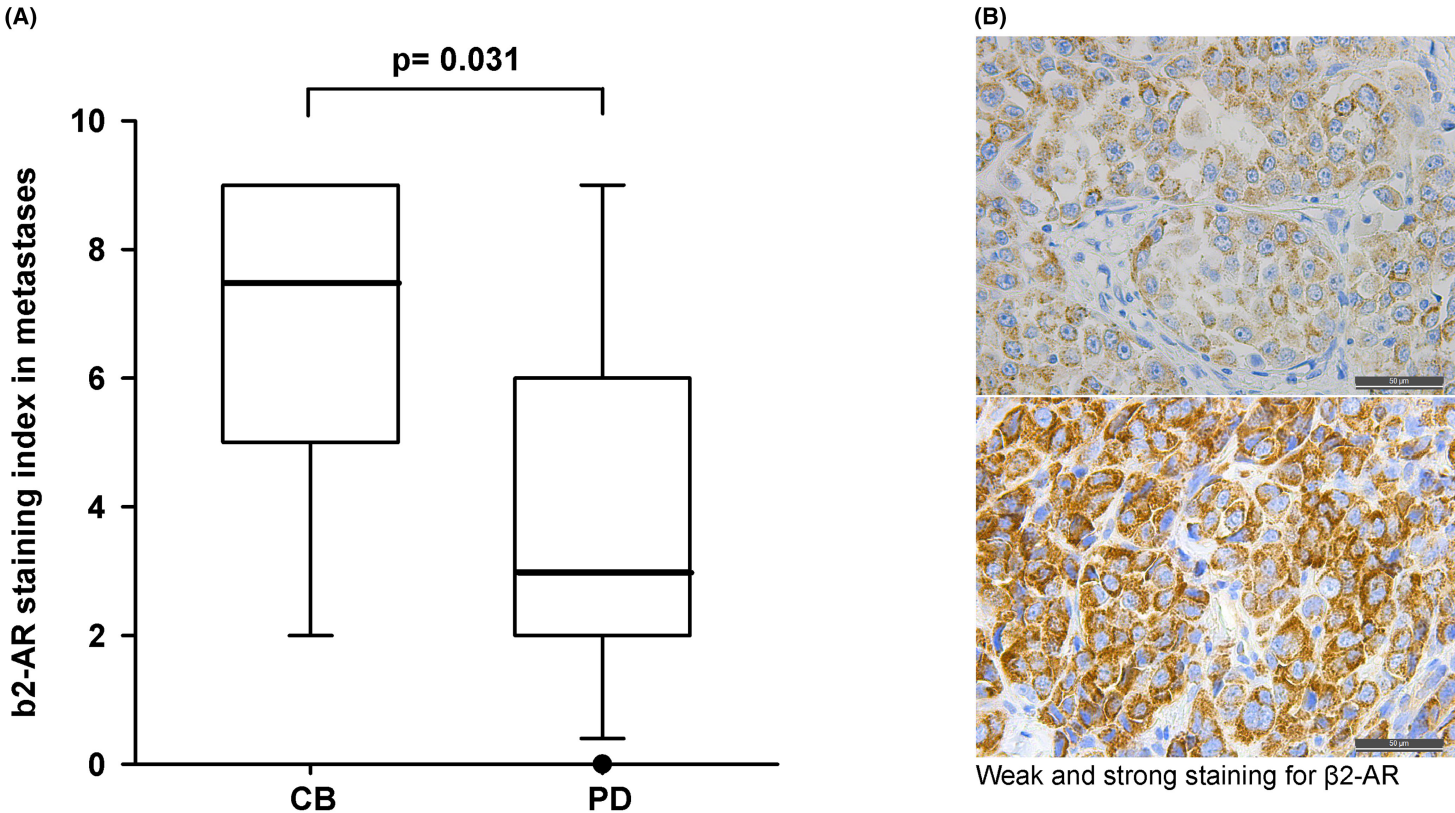
(A) Expression of β2‐adrenergic receptor in responders versus non‐responders. MWT, *p* = 0.031; CB, clinical benefit; PD, progressive disease (B) Example of weak and strong staining of β2‐adrenergic receptor in melanoma metastases.

Staining for β2‐adrenergic receptor (β2‐AR) was recorded in the cytoplasm of melanoma cells in 31/33 evaluable metastases, Figure 1B. Two cases showed negative staining. Expression of β2‐AR was significantly stronger in patients showing clinical benefit (CB) to treatment with bevacizumab compared to patients with progressive disease (PD) (median staining index 7.5 vs. 3.0, *p* = 0.031 MWT; *n* = 10 vs. 23), Figure 1A. 80% of the responding patients had at least a staining index of six in the metastatic lesion. Standard deviation was 2.8 for responders (minimum SI 2, maximum SI 9), respective 3.0 for non‐responders (minimum SI 0, maximum SI 9).

Strong β2‐AR expression was correlated with strong VEGF‐A expression in metastases (*R* = 0.495, *p* = 0.003; Spearman correlation test). The existence of TILs in metastases was not related to expression of β2‐AR (*p* = 0.246, MWT).

### 3.2 Expression of IL6‐receptor

**FIGURE 2:**
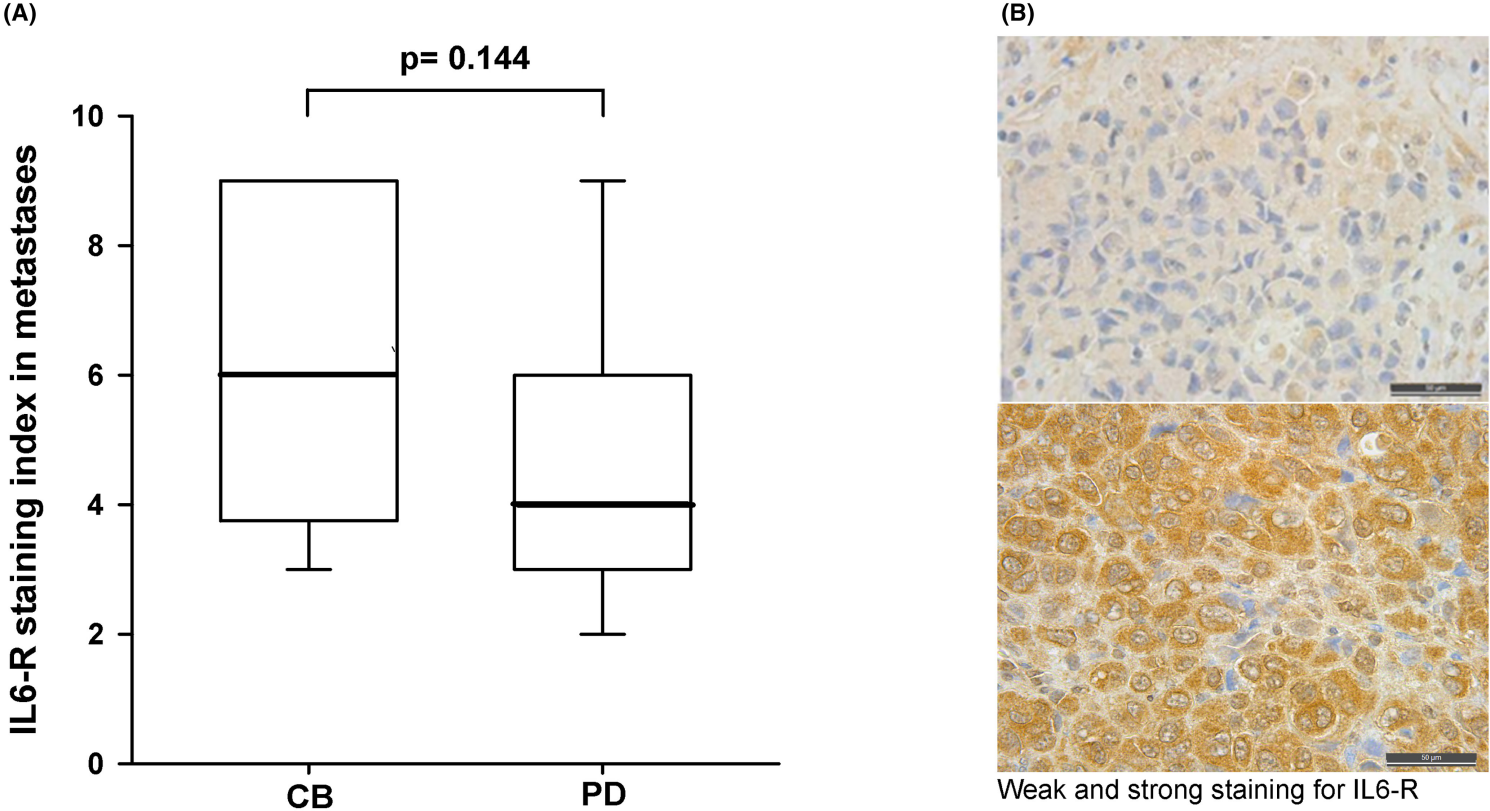
(A) Expression of IL6 receptor in responders versus non‐responders. MWT, *p* = 0.144; CB, clinical benefit; PD, progressive disease (B) Example of weak and strong staining of IL6‐receptor in melanoma metastases.

Cytoplasmatic IL6‐receptor (IL6‐R) expression was evaluable in all available metastases (33/33), Figure 2B. Expression of IL6‐R did not distinguish patients with CB from non‐responding patients, Figure 2A. The median SI was 6 in patients with CB (*n* = 10) and 4 in patients with progressive disease (*n* = 23) (*p* = 0.144, MWT). Standard Deviation for IL6‐R staining was 2.4 for responders (minimum SI 3, maximum SI 9), respective 2.3 for non‐responders (minimum SI 3, maximum SI 9). There was a trend for correlation of high IL6‐R and high β2‐AR (*R* = 0.344, *p* = 0.05; Spearman).

Presence of TILs in metastases was not associated to IL6‐R expression (*p* = 0.184, MWT). Expression of IL6‐R and VEGF‐A in metastases were not related (*p* = 0.49, Spearman).

### 3.3 Expression of cyclooxygenase‐2

**FIGURE 3:**
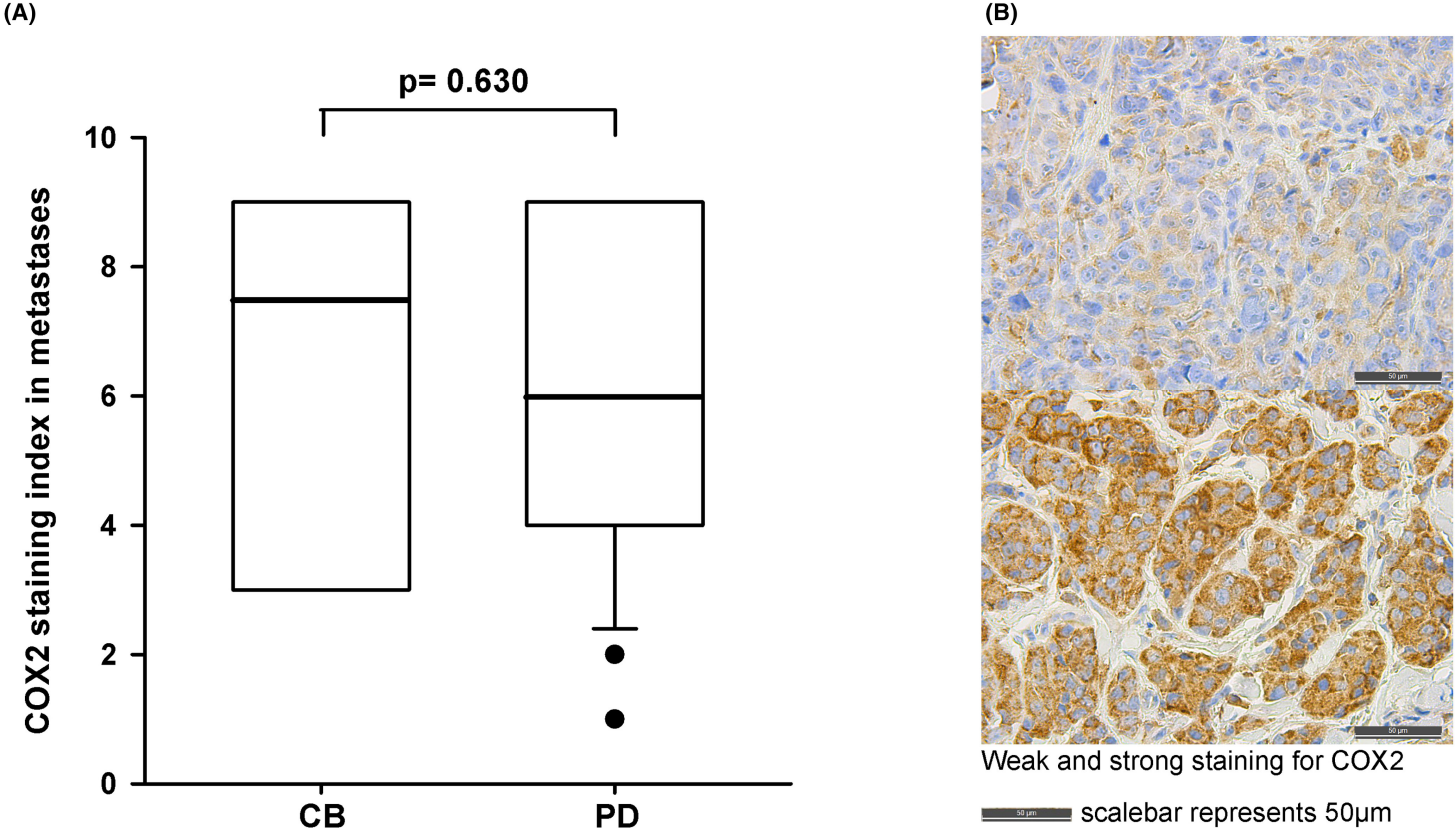
(A) Expression of COX2 in responders versus non‐responders. MWT, *p* = 0.630; CB, clinical benefit; PD, progressive disease (B) Example of weak and strong staining of COX2 in melanoma metastases.

COX2 staining was localised in the cytoplasm of all evaluable melanoma metastases (33/33), Figure 3B. Expression of COX2 in metastasis was not correlated to CB, Figure 3A. The median SI was 7.5 in the benefitting versus 6.0 in the non‐benefitting group (*p* = 0.630, MWT). Standard Deviation for COX2 staining was 2.7 in responding patients (minimum SI 3, maximum SI 9; *n* = 10) and 2.6 in non‐responding patients (minimum SI 1, maximum SI 9).

Expression of COX2 was positively associated to expression of β2‐receptor and expression of IL6‐R in metastases (*R* = 0.483, *p* = 0.004; respectively *R* = 0.535, *p* = 0.001; Spearman). Expression of COX2 and VEGF‐A in metastases was not correlated (*p* = 0.38, Spearman).

### 3.4 TILs

**FIGURE 4:**
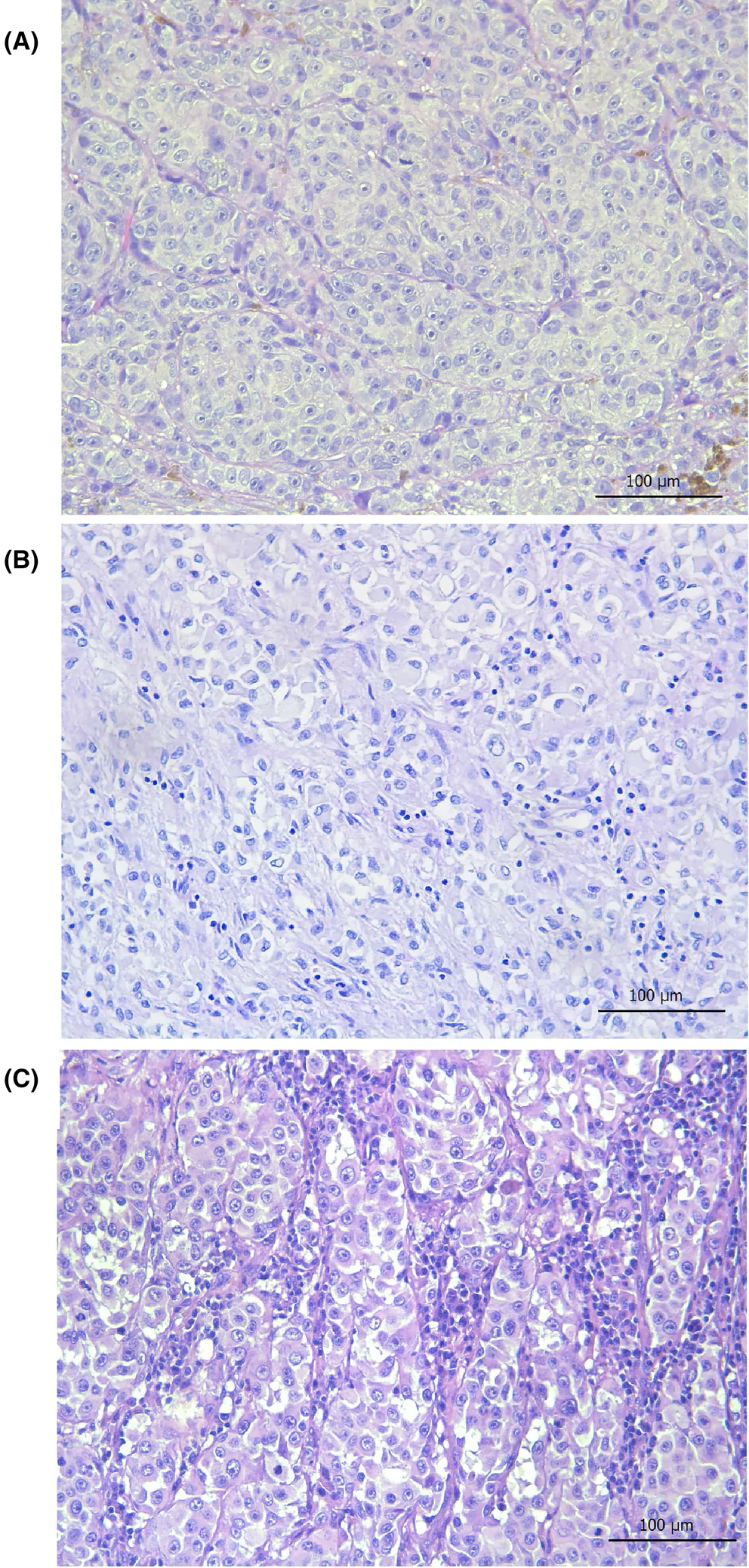
Examples of Hematoxylin and Eosin‐stained tissue samples presenting tumour‐infiltrating lymphocytes (TILs) in melanoma metastases. (A) TILs absent (B) Sample with ‘non‐brisk’ TILs (C) Sample with ‘brisk’ TILs.

Presence of TILs was assessed in 35/35 metastases. ‘Absence’ was reported in 4/35, ‘non‐brisk’ in 26/35 and ‘brisk’ in 5/35 cases. There was no association between CB and type of lymphocyte infiltration in metastases (*p* = 0.370, MWT), neither was there any correlation to response when the categories ‘TILs absent’ versus ‘TILs present’ were analysed (*p* = 0.159, *R* = −0.234, Spearman's rho). Examples for ‘absent’, ‘non‐brisk’ and ‘brisk’ lymphocyte infiltration in metastases are added in Figure 4.

## 4 DISCUSSION

Recent research highlighted the involvement of beta‐adrenergic signalling in tumour progression and immunosuppression.^12^, ^13^, ^41^ Our current findings show a significant relation between strong β2‐AR expression and clinical benefit from bevacizumab in melanoma patients with distant metastases. Since beta adrenergic signalling supports a pro‐tumorigenic immune microenvironment, it has gained increased focus in the era of immunotherapy. Additionally, research demonstrates the immune modulatory impact of VEGF, and inhibiting this pathway seems to target an immunosuppressive environment. Adrenergic signalling supports recruitment of macrophages and myeloid derived suppressor cells (MDSCs) as well as impairment of dendritic cell function.^12^, ^41^ By inhibition or silencing of β2‐AR in preclinical models, these unfavourable effects could be diminished. Consequently, tumour growth, angiogenesis and invasiveness were reduced and a shift towards a more anti‐tumorigenic TME could be induced.^17^, ^18^, ^19^, ^42^ Most models emphasised the specificity of β2‐adrenergic signalling, since initiation or inhibition of β1‐AR did not affect these mechanisms. One of the commonly applied drugs in this context is the non‐selective beta blocker propranolol. In line with preclinical findings, an increase in the ratio of CD8^+^ T cell/mMDSC as well as the percentage of CD8^+^ T‐cells was measured in responders 3 weeks after initiation of treatment with propranolol and pembrolizumab in a recent phase Ib trial.^21^ At the same time, IFNy concentration rose and the level of IL6 decreased in responding patients.^21^ The combination of these drugs also achieved a higher response rate than historically reported for pembrolizumab monotherapy.^22^ Accordingly, better outcome was also reported among melanoma patients using beta‐blockers concomitant when treated with immunotherapy.^20^ Overall, preclinical and clinical data support an importance of β2‐adrenergic signalling in tumour cells and the TME. Lastly, inferior outcome is reported in case of increased β2‐AR expression in colorectal cancer^43^ and adenocarcinoma of the lungs.^44^ Altogether, these findings support that enhanced expression of β2‐AR may represent an unfavourable characteristic in melanoma metastasis.

Moreover, we examined the association of β2‐AR expression to the expression of VEGF‐A, COX2 and IL6‐R. The expression of β2‐AR was positively correlated with VEGF‐A and COX2 expression in melanoma metastasis and a trend for correlation between β2‐AR and IL6‐R expression was observed. Preclinical models provide evidence that β2‐AR signalling affects the regulation of VEGF‐A as well as other angiogenic and inflammatory cytokines.^14^, ^25^ These models provide evidence for the impact of beta‐adrenergic signalling on VEGF‐A and IL6 in melanoma cells due to altered expression after stimulation with catecholamines.^14^, ^25^ In line with this, levels of VEGF‐A and IL‐6 were increased in a β‐AR expressing breast cancer cell line^26^ and ovarian cancer cells.^18^ These preclinical findings support an association between increased expression of β2‐AR and VEGF‐A in our material. Recent data emphasised VEGF‐A as an immunomodulator beside its role as an important pro‐angiogenic factor.^5^ VEGF impairs dendritic cell function and recruitment of CD8^+^ T cells, and additionally promotes aggregation of myeloid‐derived suppressor cells and regulatory T‐lymphocytes and supports a shift towards Type 2 macrophages. Anti‐VEGF or anti‐VEGFR treatment abolished the immunosuppressive effects of VEGF.^5^ Our data suggest that treatment with bevacizumab reduces unfavourable immune modulating effects of VEGF‐A and beta‐adrenergic signalling as well as the interplay between them. This is reflected by the correlation between β2‐AR expression and treatment benefit in our material.

Further, our findings show an association between COX2 and β2‐AR expression in melanoma metastasis. COX2 supports tumour progression, among others by immune escape,^29^, ^32^, ^33^ and its expression can be modulated by beta adrenergic signalling.^45^ Importantly, COX2 is normally not expressed in most human tissues but its expression is upregulated during inflammation and tumorigenesis.^29^ Additionally, COX2 directly impairs maturation and function of dendritic cells and promotes proliferation of M2 macrophages.^33^ In conclusion, activation of COX2 results in a more pro‐tumorigenic TME that favours immune escape and tumour progression. Similar mechanisms are upregulated by β‐adrenergic signalling and possibly, there is an interplay between these pathways. To speculate, our results might suggest that increased β2‐AR signalling contributes to the immune suppressing effects of VEGF in melanoma, and sensitises tumour cells to the efficacy of specific VEGF‐A blockade by bevacizumab.

As mentioned previously, there is no clear consensus about the prognostic and predictive value of TILs assessed in H&E sections and outcome in melanoma patients. However, most studies have focused on TILs in primary melanoma. When Hamid et al. investigated TILs in metastasis at baseline and during treatment with ipilimumab, TILs at baseline were not correlated to response.^46^ In our series, TILs were present in 31/35 metastases. In line with Hamid, the presence of TILs in metastasis did not predict CB to bevacizumab in our material. Core biopsies or fine needle aspirations contain only a small amount of tumour tissue thereby providing only an incomplete picture of the lesion. The prognostic and predictive value of TILs in metastasis should therefore be evaluated carefully.

The advantage of our study is the application of bevacizumab as monotherapy in metastatic melanoma. Treatment with a single agent allows to investigate the direct effects of this specific drug. Since all patients are followed at our department, the clinical data are complete and regularly updated over a period of 12 years. Despite our intriguing results, the limited number of patients in this single arm study highlights the need for validation in a larger sample size, preferable in a randomised trial. At the time of enrolment, immune checkpoint blockade was yet to be implemented and no available treatment translated into significantly improved OS. Thus, investigating the efficacy of bevacizumab monotherapy, and exploring potential predictive markers was considered as a valuable option. Further, this study lacks a parallel preclinical translational part to perform basic mechanistic studies on patient samples. Lastly, the amount of tumour tissue is limited in some core needle biopsies and might not capture the distribution of our markers of interest completely. However, tissue sampling was performed according to established practice.

In conclusion, to the best of our knowledge, we are the first to introduce β2‐AR in melanoma metastasis as a new candidate biomarker for response to anti‐VEGF‐A treatment. This finding is of special interest since recent research has confirmed the influence of VEGF‐A signalling as well as adrenergic signalling on immune response mechanisms. Upregulation of these signalling pathways supports tumour progression by favouring an immunosuppressive microenvironment. In that context, the role of β2‐AR as a predictive marker should be further investigated in treatment of malignant melanoma with immune checkpoint blockade. Furthermore, expression of β2‐AR potentially characterises a certain subgroup of patients who could derive therapeutic benefits from the combined use of anti‐VEGF‐A treatment and ICB. Additionally, our data encourage trials adding beta‐blockers to the combination of anti‐VEGF‐A treatment and ICB. By doing so, the unfavourable tumour microenvironment can be altered leading to improved responsiveness to ICB.

## AUTHOR CONTRIBUTIONS

**Cornelia Schuster:** Conceptualization (lead); data curation (lead); formal analysis (lead); investigation (lead); methodology (equal); visualization (lead); writing – original draft (lead); writing – review and editing (equal). **Lars A Akslen:** Conceptualization (supporting); data curation (supporting); formal analysis (supporting); funding acquisition (lead); methodology (equal); writing – review and editing (equal). **Oddbjørn Straume:** Conceptualization (equal); data curation (supporting); formal analysis (supporting); funding acquisition (equal); investigation (supporting); methodology (equal); project administration (lead); supervision (supporting); validation (supporting); visualization (supporting); writing – review and editing (equal).

## FUNDING INFORMATION

This project received partial funding from the Research Council of Norway via its funding initiative for Centers of Excellence, with the assigned identification number 223250.

## CONFLICT OF INTEREST STATEMENT

The authors have no conflict of interests to declare.

## ETHICS STATEMENTS

Approval by the Regional Ethics Committee (processing number: 05/329) and the Norwegian Medicines Agency was obtained, and the project was performed according to the ethical principles of the Declaration of Helsinki and the International Conference on Harmonisation of Good Clinical Practice. Informed consent was signed by all participants before enrolment.

## TRIAL REGISTRATION NUMBER

NCT00139360.

## Supporting information


Data S1.

## Data Availability

Relevant data are provided in the manuscript. Further data that support the findings of this study are available on request from the corresponding author. The data are not publicly available due to privacy or ethical restrictions.
