# Supplementary material for: β2‐adrenergic receptor expression in patients receiving bevacizumab therapy for metastatic melanoma
Source: Cancer Med. 2023 Aug 8;12(17):17891–900. doi: 10.1002/cam4.6424 (PMC10524038; doi:10.1002/cam4.6424)
Supplement: Supplementary file 1 — Data S1. [file CAM4-12-17891-s001.docx]

**Suppl.** **Table 1** Inclusion and exclusion criteria

| **Inclusion criteria** | **Exclusion criteria** |
| --- | --- |
| Histologically confirmed unresectable metastatic melanoma in progression | Brain metastases |
| Age >18 years | Absolute neutrophils <1.0×109/L |
| WHO performance status 0–2 | Platelets < 100×109/L |
| Clinically and/or radiographically measurable disease according to RECIST | Bilirubin, creatinine, INR > 1.5× upper normal limit |
| > 4 weeks since adjuvant interferon | Symptomatic congestive heart failure, angina pectoris or cardiac arrhythmia |
| No prior interferon or interleukin for metastatic disease | History of thrombosis |
| Recovered from prior chemotherapy | Uncontrolled hypertension |
| No major surgery within 28 days | Full dose coumarin-derived anticoagulants or NSAIDS |
| Able to undergo outpatient treatment | Pregnant or lactating patients |

Ref: Clinical Efficacy and Safety of Bevacizumab Monotherapy in Patients with Metastatic Melanoma: Predictive Importance of Induced Early Hypertension. Schuster et al 2012

**Suppl. Table 2** Histopathologic features in hematoxylin and eosin-stained sections of primary tumours

| Histologic type by Clark |
| --- |
| Tumor thickness by Breslow |
| Mitotic rate |
| Ulceration |
| Necrosis |
| Clark’s level of invasion |
| Growth phase |
| Tumour infiltrating lymphocytes |
| Vascular invasion |

**Suppl. Figure 1**


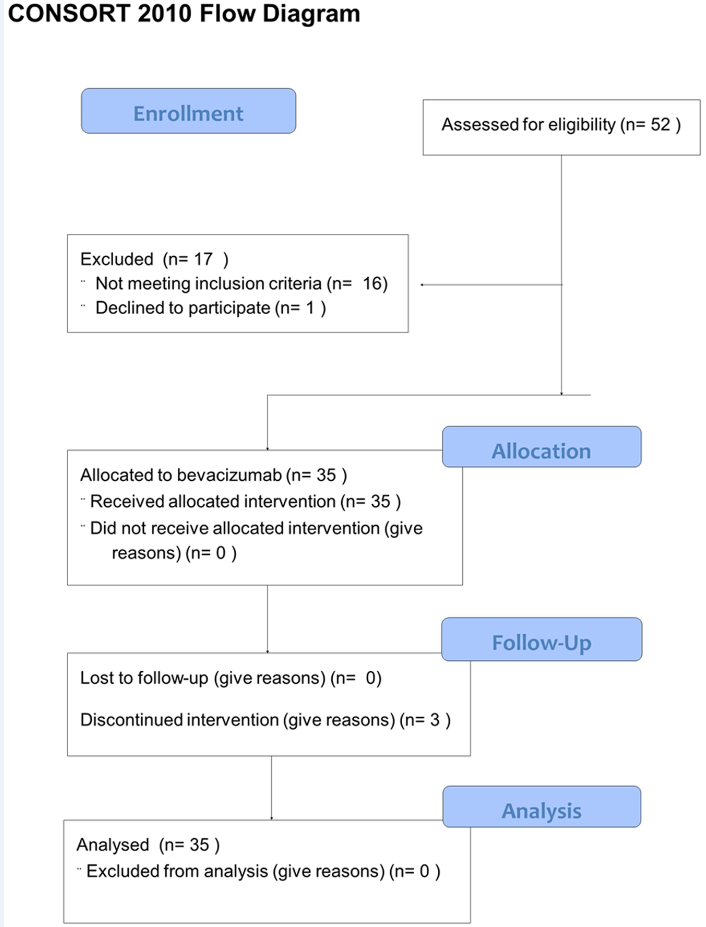


Ref: Clinical Efficacy and Safety of Bevacizumab Monotherapy in Patients with Metastatic Melanoma: Predictive Importance of Induced Early Hypertension. Schuster et al 2012

**Suppl. Figue 2**


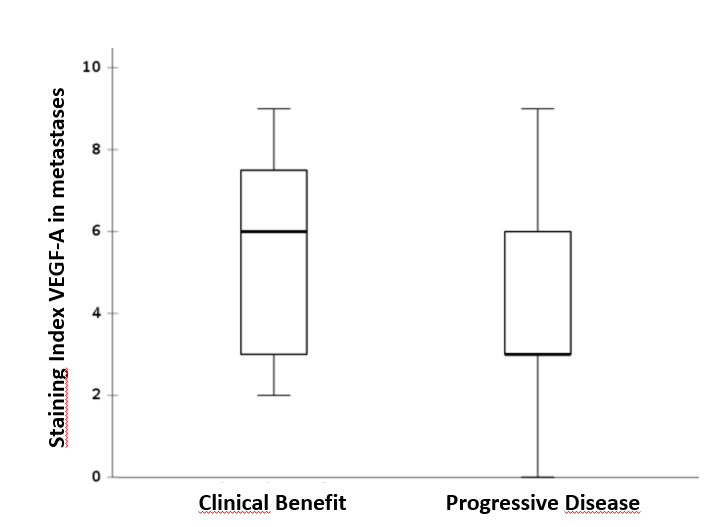


Ref: Expression of Heat Shock Protein 27 in Melanoma Metastases Is Associated with Overall Response to Bevacizumab Monotherapy: Analyses of Predictive Markers in a Clinical Phase II Study. Schuster et al 2016
